# The Sonodegradation of Caffeic Acid under Ultrasound Treatment: Relation to Stability

**DOI:** 10.3390/molecules18010561

**Published:** 2013-01-04

**Authors:** Yujing Sun, Liping Qiao, Xingqian Ye, Donghong Liu, Xianzhong Zhang, Haizhi Huang

**Affiliations:** 1 Department of Food Science and Nutrition, School of Biosystems Engineering and Food Science, Zhejiang University, Hangzhou 310058, China; 2 Fuli Institute of Food Science, Zhejiang University, Hangzhou 310058, China

**Keywords:** ultrasound, caffeic acid, stability, kinetics, degradation

## Abstract

The degradation of caffeic acid under ultrasound treatment in a model system was investigated. The type of solvent and temperature were important factors in determining the outcome of the degradation reactions. Liquid height, ultrasonic intensity and duty cycle only affected degradation rate, but did not change the nature of the degradation. The degradation rate of caffeic acid decreased with increasing temperature. Degradation kinetics of caffeic acid under ultrasound fitted a zero-order reaction from −5 to 25 °C. Caffeic acid underwent decomposition and oligomerization reactions under ultrasound. The degradation products were tentatively identified by FT-IR and HPLC-UV-ESIMS to include the corresponding decarboxylation products and their dimers.

## 1. Introduction

Caffeic acid and its analogues are widely distributed in the plant kingdom and are found in coffee beans, olives, propolis, fruits, and vegetables [1,2,3]. They are usually found as various simple derivatives including amides, esters, sugar esters, and glycosides, or in rather more complex forms such as rosmarinic acid (dimer), lithospermic acid (trimer), verbascoside (heterosidic ester and glycoside of dihydroxyphenethylethanol and caffeic acid), and the flavonoid-linked derivatives. They possess antioxidant activities [4] and antibacterial activity [5], and also can reduce the incidence of tumors [6] and cardiovascular diseases [2].

However, caffeic acid and its analogues are sensitive to degradation due to the nature of their chemical structures (such as the dihydroxyl in the catechol ring, and carboxyl and ethylene group in the side chain) and the external conditions (such as heat, light, irradiation and so on). Previous studies have reported the impact of food processing on the stability of caffeic acid and its analogues. For example, chlorogenic acid, caffeic acid and cinnamic acid in apple juice suffered degradation during the ozonation of cloudy apple juice [7]. Roasting of coffee resulted in the degradation of chlorogenic acid [8]. In roasted coffee, caffeic acid, quinic acid and chlorogenic acid are degraded to volatiles, and the reaction of catechol from caffeic acid under anaerobic conditions was not oxidative degradation [9,10]. Meanwhile, the degradation of caffeic acid may also occur during the extraction from fruits and vegetables. For example, gentisic acid, gallic acid, *p*-hydroxybenzoic acid, vanillic acid, caffeic acid, *p*-coumaric acid, ferulic acid and sinapic acid are stable up to 100 °C, whereas at 125 °C there was significant degradation during microwave-assisted extraction [11].

Ultrasound-assisted extraction has been widely used for the extractions of bioactive compounds due to the high extraction efficiency and extraction rate [12,13,14], but the cavitation effect of ultrasound may accelerate or trigger chemical reactions in the extraction medium. For example, ultrasound treatment caused the degradation of flavonoids [15] and carotenoids [16], and the aggregation and decomposition of polysaccharides [17,18], but the sonochemical effects on caffeic acid under ultrasound power are seldom reported. Ma, *et al*. [19] found that the extraction yields of total phenolic acids from citrus peel decreased with extended time under a relatively higher temperature. Nikolopoulos, *et al*. [20] found that the combined ultrasonic/catalytic process can decompose 4-hydroxybenzoic acid in olive mill wastewater and is very promising for environmental applications. However, the stability of caffeic acid under ultrasound treatment remains unclear. The objective of this study was to determine the effects of different factors of ultrasound treatment on the stability of caffeic acid in a model system, the kinetics and products of degradation. The results should help to understand and control the degradation of caffeic acid during ultrasound treatment.

## 2. Results and Discussion

### 2.1. Effect of Solvent on the Stability of Caffeic Acid

**Figure 1:**
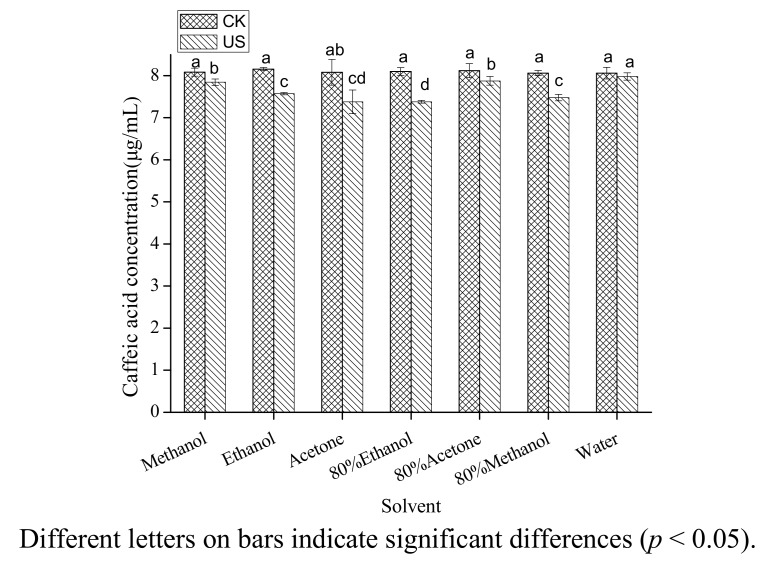
Effect of solvent on the stability of caffeic acid under ultrasound treatment (US) and maceration (CK).

The effect of different solvents on the concentration of caffeic acid treated by ultrasound is shown in Figure 1. The seven polar solvents (methanol, ethanol, acetone, 80% ethanol, 80% acetone, 80% methanol, water) examined in the present study are among those most often used for the extraction of phenolic acids in the traditional extraction methods [21,22,23,24]. Considering the extraction yields of phenolic acids, mixtures of alcohol (methanol or ethanol)-water or acetone-water are the best extraction solvents [21], but pure organic solvent (methanol) may be the best extraction solvent under ultrasound treatment considering the stability of caffeic acid. Caffeic acid degraded significantly (*p* < 0.05) in all the solutions and the degradation rates in the seven tested solvents were significantly different. Compared with the initial concentration, caffeic acid was reduced by 8.90% under ultrasound treatment in 80% ethanol and in water it was only decreased by 1.02%. It could thus be concluded that ultrasound caused much stronger chemical effect on caffeic acid in 80% ethanol than in other solvents.

Mixtures of ethanol and water are the most popular solvents to extract phenolics because of their green characteristics and high extraction yields [22]. However, in the present study we found that caffeic acid had poor stability in 80% ethanol under ultrasound treatment. To explain the special phenomena, the following experiments were carried out in 80% ethanol.

### 2.2. Effect of Temperature on the Stability of Caffeic Acid

**Figure 2:**
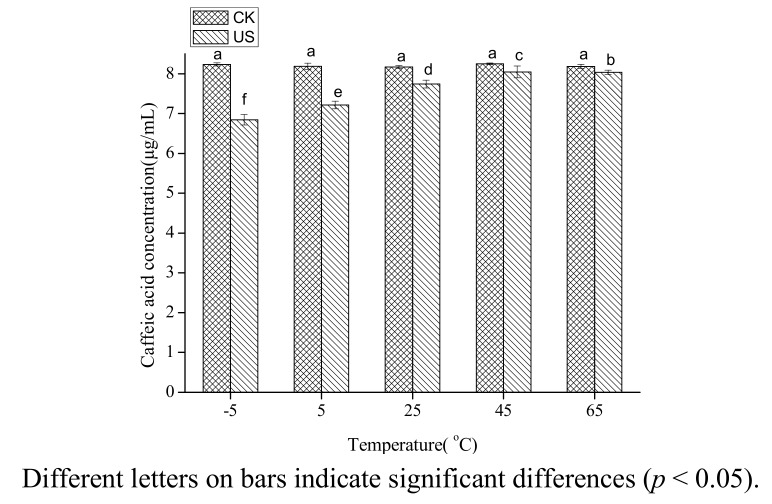
Effect of temperature on the stability of caffeic acid under ultrasound treatment (US) and maceration (CK).

Caffic acid in 80% ethanol was treated by ultrasound at −5, 5, 25, 45, 65 °C. The range of temperatures studied was less than the thermal degradation temperature of caffeic acid. The degradation of caffeic acid in 80% ethanol decreased with increasing temperature (*p* < 0.05) (Figure 2). The concentration of caffeic acid under ultrasound treatment was 83% of the untreated at −5 °C, and was 98% of the untreated at 65 °C.

The results may be explained by the decreasing cavitation intensity with increasing temperature. The physical properties (surface tension, viscosity, vapour pressure) of the solvent are the main factors affecting cavitation intensity, and the most important factor among these properties is the vapour pressure [25,26]. Vapour pressure of a solvent is negatively correlated with cavitation intensity and increases with increasing temperature. Therefore, cavitation intensity decreases with increasing temperature. These results are in agreement with the previous finding that the cavitation intensity decreased with an increase in temperature [16]. Romdhane, *et al*. [27] also found that the oxidation rate of aqueous potassium iodide under ultrasound decreased with increasing temperature. It was inferred that the sonochemical reactions would not fit Arrhenius theory.

### 2.3. Effect of Liquid Height on the Stability of Caffeic Acid

**Figure 3:**
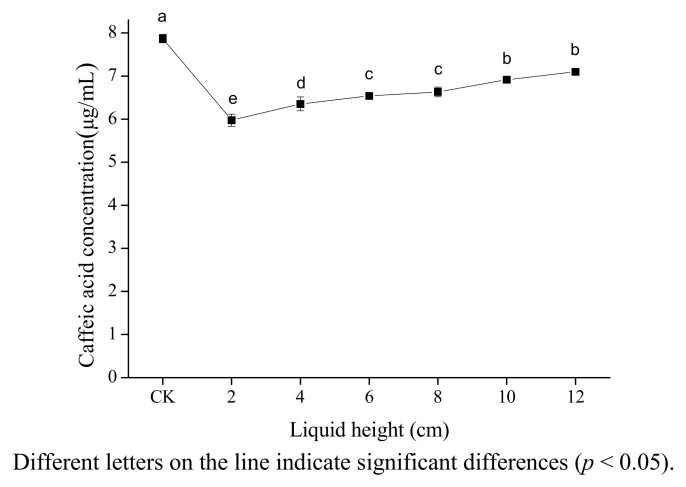
Effect of liquid height on the stability of caffeic acid under ultrasound treatment (US).

Figure 3 shows on caffic acid concentration in 80% ethanol of liquid height measured the distance from the horn microtip to tube bottom. From the figure, it can be seen that caffeic acid concentration increased significantly with height ranging from 2 to 12 cm. (*p* < 0.05). This may be due to the fact that the cavitation intensity decreases with increasing height because of the attenuation of the waves caused by absorption and scattering. Other authors have also found that the maximum ultrasound power was observed in the vicinity of the radiating surface of the ultrasonic horn, and that ultrasonic intensity decreased rather abruptly as the distance from the radiating surface increases [28,29]. This is different from the result of Sun [16] whereby β-carotene concentration decreased markedly under ultrasound with height ranging from 2 to 6 cm, then increased slightly with height ranging from 6 to 12 cm. The difference may be caused by the differences of attenuation coefficients of two solutions.

### 2.4. Effect of Electrical Ultrasonic Intensity on the Stability of Caffeic Acid

**Figure 4:**
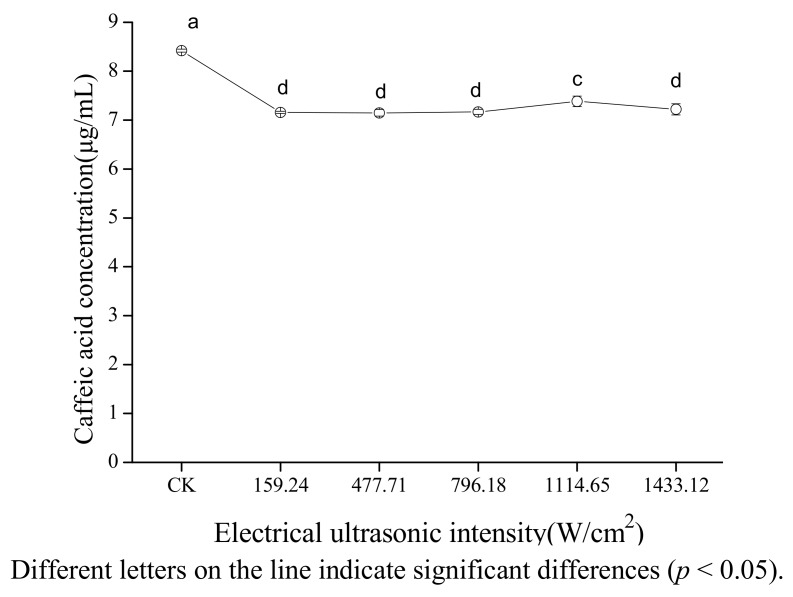
Effect of electrical ultrasonic intensity on the stability of caffeic acid under ultrasound treatment (US).

Figure 4 shows the effect of electrical ultrasonic intensity on the concentration of caffeic acid in 80% ethanol. The concentration of caffeic acid did not change significantly with the increasing electrical ultrasonic intensity ranging from 159.24 to 796.18 W/cm^2^, increased with the increasing electrical ultrasonic intensity ranging from 796.18 to 1,114.65 W/cm^2^ (*p* < 0.05), and decreased slightly when the electrical ultrasonic intensity ranged from 1,114.65 to 1,433.12 W/cm^2^. The reason for this could be that it is easier to form cavitation bubbles, and the bubbles collapsed more violently with increasing electrical ultrasonic intensity in the range of from 159.24 to 796.18 W/cm^2^ [30]. When the electrical ultrasonic intensity ranged from 796.18 to 1,114.65 W/cm^2^, the cavitation bubbles may grow too big to collapse or collapse weakly which caused a reduction in the cavitation effect. Also the presence of many bubbles may have hampered the propagation of the ultrasound waves [31]. The slightly decrease of concentration of caffeic acid may be due to the combination of reduction of cavitation effect and the absorption of heat as electrical ultrasonic intensity ranged from 1,114.65 to 1,433.12 W/cm^2^. The absorption of heat can be explained by the fact that the heat caused by ultrasound was dissipated completely in a short time when the electrical ultrasonic intensity is less than 1,114.65 W/cm^2^; however, the heat caused by ultrasound was not dissipated completely in a short time when electrical ultrasonic intensity is greater than 1,114.65 W/cm^2^ because of the increasing of heat with ultrasonic intensity.

### 2.5. Effect of Duty Cycle of Ultrasonic Exposure on the Stability of Caffeic Acid

**Figure 5:**
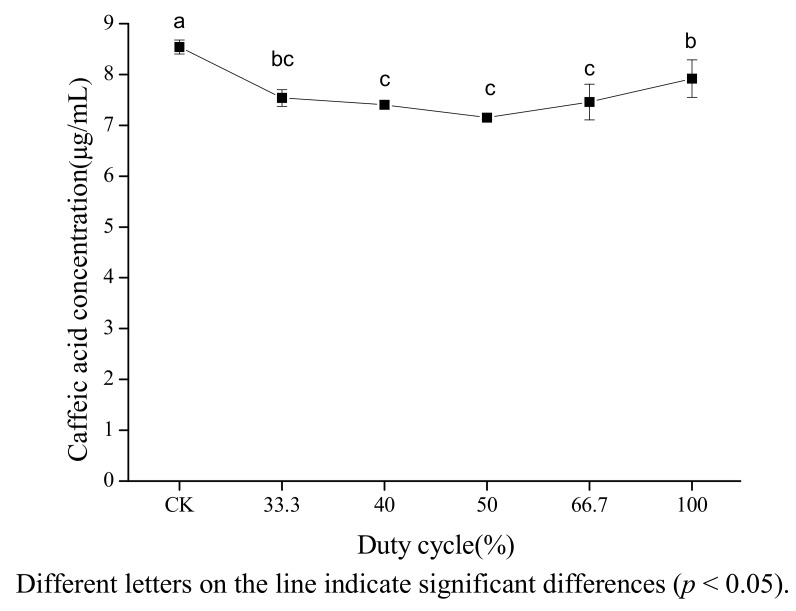
Effect of duty cycle on the stability of caffeic acid under ultrasound treatment (US).

The effect of duty cycle on the concentration of caffeic acid in 80% ethanol was investigated at a width of pulse of 2 s. Figure 5 shows that both pulsed and continuous ultrasound decreased the concentration of caffeic acid. The degradation rate of caffeic acid under pulsed ultrasound was higher than under continuous ultrasound. However, no clear explanation can be provided. The effect of duty cycle on cavitation effect in different literature reports was inconsistent. Luque-García [20] found that duty cycle was not the major factor in the extraction of total fat from oleaginous seeds. Sun [16] found that β-carotene had the highest degradation rate when the duty cycle was 66.7%.

### 2.6. Degradation Kinetics of Caffeic Acid under Ultrasound Treatment

**Figure 6:**
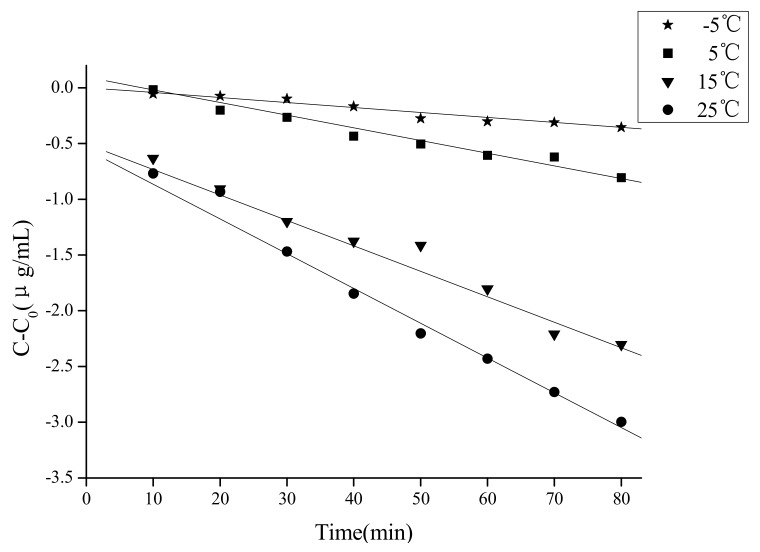
Degradation kinetics curves of caffeic acid under ultrasound treatment (US) at −5, 5, 15, 25 °C.

In the present study we only investigated the kinetics of caffeic acid under ultrasound from −5 to 25 °C because of the weak degradation observed from 25 to 65 °C. The corresponding results of determination coefficients (R^2^) between c, lnc, 1/c, 1/c^2^ and time at −5, 5, 15, 25 °C are summarized in Table 1. According to the trial-and-error procedure, the degradation kinetics of caffeic acid in 80% ethanol under ultrasound fitted a zero-order reaction equation at −5 to 25 °C. The kinetics curves from −5 to 25 °C are presented in Figure 6, respectively. The concentration of caffeic acid was proportional to treatment time at −5 to 25 °C. Kinetic parameters k, R^2^, t1/2, were calculated according the models at each temperature (Table 2). It also can be seen that the degradation rate of caffeic acid was much higher at low temperature than high temperature by comparing of the *k* and t_1/2_. For example, the degradation rate of caffeic acid at −5 °C was 7 times of the degradation rate at 25 °C.

**Table 1:** Correlation coefficients (R) of C, lnC, 1/C, 1/C2 of caffeic acid and time at −5, 5, 15, 25 °C under ultrasound treatment.

T (°C)	R Zero(C)	R First(lnC)	R Second (1/C)	R Third (1/C^2^)
−5	0.974	0.98	0.983	0.983
5	0.932	0.929	0.924	0.918
15	0.915	0.916	0.917	0.917
25	0.956	0.957	0.957	0.957

**Table 2:** Degradation Kinetics Parameters *k* (rate constant), R^2^ (determination coefficients), t_1/2_(half-life periods) of caffeic acid under ultrasound treatment.

T (°C)	*k*(ug mL^−1^ min^−1^) (*p* < 0.05)	R^2^ (*p* < 0.05)	t_1/2_ (min)
−5	0.0331	0.949	166.163
5	0.0239	0.869	230.126
15	0.0104	0.837	528.846
25	0.00475	0.914	1157.895

### 2.7. Degradation Products Analysis

**Figure 7:**
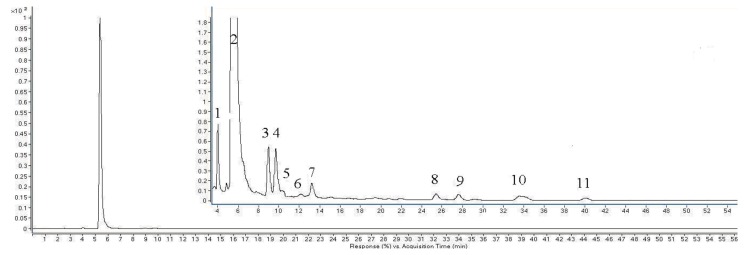
HPLC chromatogram of caffeic acid treated by ultrasound at −5 °C.

The HPLC-DAD chromatographic peaks and FT-IR spectra of caffeic acid treated by ultrasound from −5 to 25 °C were similar, so we only listed the degradation products of caffeic acid at −5 °C. Several new chromatographic peaks appeared in the HPLC-DAD traces after caffeic acid was treated by ultrasound (Figure 7). The corresponding ESIMS spectral data of the degradation compounds from caffeic acid are shown in Table 3.

**Table 3:** Identification of new chromatographic peaks in HPLC-DAD after caffeic acid treatment by ultrasound.

Peak No.	HPLC tR(min)	Molecular weight	HPLC-ESIMS(*m/z*)	HPLC+ESIMS(*m/z*)	Tentative identification
1	3.97	138	137.1	139.0	C_8_H_10_O_2_
2	5.36	180	179.1	163.0;181.0	Caffeic acid std
3	8.94	492	491.1	514.9	Undentified
4	9.66	220	219.1	220.8; 243.0	Undentified
5	10.23	358	357.1; 393.1	359.0; 381.0	Caffeic dimer
7	13.20	160	-	161.0; 183.0	Undentified

The ESIMS spectrum of Peak 1 showed the [M–H]^−^ molecular ion at *m/z* 137.1 and the [M+H]^+^ one at *m/z* 139.0, therefore the molecular weight of this compound was established as 138 (C_8_H_10_O_2_). It may be the result of decarboxylation of the caffeic acid matrix (180 – 44 + 2H = 138). For Peak 2, its retention time of HPLC (5.28 min) and mass spectral peaks ([M–H]^−^ at *m/z* 179.1, [M+H]^+^ at *m/z* 181.0, [M-H_2_O+H]^+^ at *m/z* 163.0) are identical to those of a caffeic acid standard, thus Peak 2 was unambiguously identified as caffeic acid. The molecular weight of Peak 3 was 492 according to its [M–H]^−^ peak at *m/z* 491.1 and [M+Na]^+^ at *m/z* 514.9. The molecular weight of Peak 4 was 220 based on a [M–H]^−^ at *m/z* 219.1, [M+H]^+^ at *m/z* 220.8 and [M+Na]^+^ at *m/z* 243.0. However, a reasonable explanation of Peaks 3-4 could not be given based on the molecular weights and our knowledge of caffeic acid, which needs further investigation. With evidence of four molecular ions [M–H]^−^ at *m/z* 357.1, [M+Cl]^−^ at *m/z* 393.1, [M+H]^+^ at *m/z* 359.0 and [M+Na]^+^ at *m/z* 381.0, the molecular weight of Peak 5 was 358, which is twice the molecular weight of caffeic acid (180 × 2 − 2H = 358), thus it was provisionally assumed to be a caffeic acid dimer. In terms of Peak 6, 8, 9, 10 and 11, the ESIMS spectra responses were too low to distinguish them clearly from the background. 

**Figure 8:**
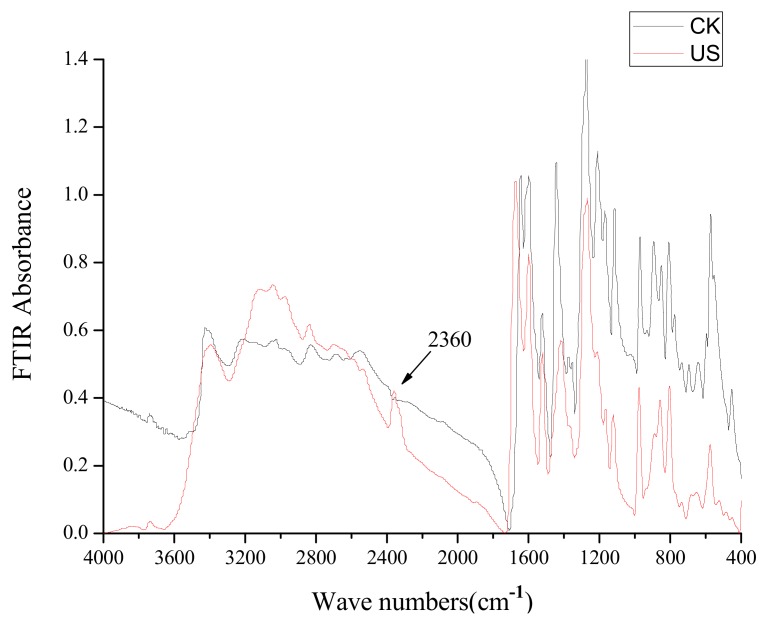
FT-IR spectra at different wave numbers (cm^−1^) corresponding to caffeic acid treated by ultrasound (US) at −5 °C.

The molecular weight of peak 7 was 160 according to the [M+H]^+^ molecular ion at *m/z* 161.0 and the [M+Na]^+^ one at *m/z* 183.0, but a reasonable structure cannot be proposed at this time. The FT-IR spectra of untreated caffeic acid treated and caffeic acid treated by ultrasound at −5 to 25 °C were different (Figure 8). A new vibration peak at 2,360 cm^−1^ corresponding to ν(CO_2_) was observed in FT-IR spectra of caffeic acid treated by ultrasound (Figure 8), suggesting that a decarboxylation reaction of caffeic acid occurred under ultrasound treatment at −5 to 25 °C.

**Figure 9:**
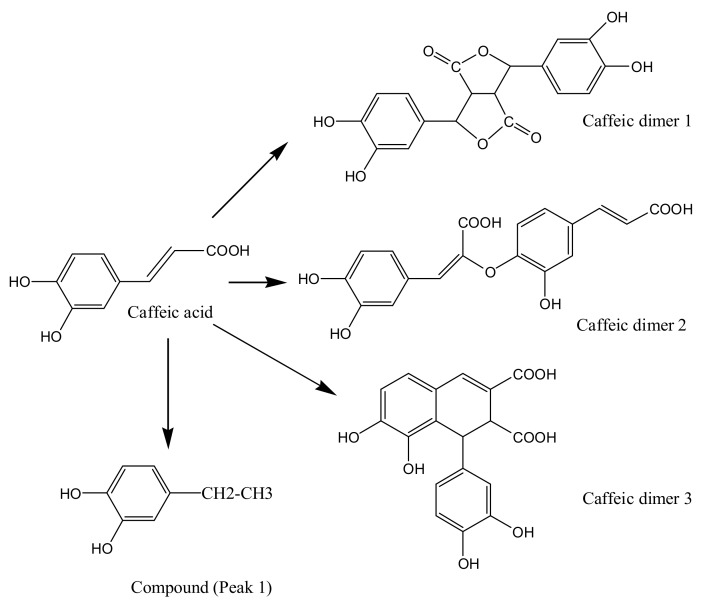
The proposed degradation mechanism of caffeic acid under ultrasound treatment (US).

From the ESIMS spectral data and the FT-IR spectra of caffeic acid treated by ultrasound, decomposition (decarboxylation) and oligomerization reactions occurred. The proposed degradation mechanism of caffeic acid under ultrasound is demonstrated in Figure 9. In the present study, we only analyzed the molecular weight and functional groups of the degradation products. The accurate structures of degradation products will be further identified in detail.

## 3. Experimental

### 3.1. Chemicals

HPLC-grade methanol was purchased from Tedia Company, Inc. (Fairfleld, OH, USA). Ethanol, acetone and acetic acid (analytical grade) were purchased from Sinopharm Chemical Reagent Co. (Shanghai, China). The standard caffeic acid (purity ≥ 98.0%) was purchased from Sigma Chemical Co. (St. Louis, MO, USA).

### 3.2. Ultrasound Treatment

Ultrasound treatments (US) were carried out with a probe ultrasonic processor (JY92-IIDN, Ningbo Scientz Biotechnology Co., Ningbo, China). Some parameters of the probe ultrasonic processor are as follows: the highest power is 900 W, frequency is 20 kHz, and the diameter of horn microtip is 6 mm. The 8.0 μg/mL solution of standard phenolic acid was prepared using volumetric flask. The solution was added to brown glass tubes (3 cm diameter × 10–20 cm height), and then the tubes with solution were immersed in low-temperature thermostatic ethanol (T ≤ 0 °C) or water (T ≥ 5 °C) bath (DC-1006, Safe Corporation, Ningbo, China) to maintain a constant temperature. The solution was treated with ultrasound. Apart from the special ultrasound conditions mentioned in the results, the general ultrasound conditions were: the probe was placed 1 cm from the top surface of the extraction cell, the liquid height measured the distance from the horn microtip to tube bottom was 4 cm, and the temperature of −5 °C (except for water 5 °C, pulsed mode (2 s on and 2 s off), the treatment time of 60 min, electrical ultrasonic intensity of 796.18 W/cm^2^. The sample macerated under the same conditions was used as control check (CK). The US and CK solution were filtered through a 0.45 μm polyvinylidene fluoride microfiltration membrane (Shanghai Xingya Purification Material Co., Shanghai, China), then stored at −18 °C for further HPLC analysis.

### 3.3. Calculation of Electrical Ultrasonic Intensity

The electrical acoustic intensity dissipated from the probe microtip was calculated according to the following formula [16]:

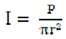
2
where r is the radius of the probe microtip, and P is the electrical input power. In the present study the input power levels was adjusted to 5%, 15%, 25%, 35%, 45% of total electrical input power (900 W) which was equal to 45, 135, 225, 315, 405 W, respectively. The corresponding electrical ultrasonic intensity were 159.24, 477.71, 796.18, 1,114.65, 1,433.12 W/cm^2^, respectively.

### 3.4. Analytical Method of Phenolic Acids

Phenolic acids UFLC analyses were carried out on an LC-20 (Shimadzu) instrument linked simultaneously to a SPD-M20A. One μL treated phenolic acid solution was injected on a reversed phase 2.0 × 100 mm, 2.9 μm CAPCELL PAK C18 MG S3 column (Shiseido Co. Tokyo, Japan). The column thermostat was set at 40 °C. Solvent A was 4% acetic acid/water, solvent B was methanol (A:B = 80:20) and flow rate was 0.2 mL/min, which was in accordance with Xu [32] with some revision. Caffeic acid was monitored at 320 nm. Caffeic acid was quantified with external standards using UFLC analysis. The concentration of caffeic acid was expressed as micrograms per milliliter solution volume (μg/mL). Standard stock solutions with varying caffeic acid concentrations were prepared. Within the range of 1–12 μg/mL the equation of linear regression was good with R^2^ > 0.997 for caffeic acid. The repeatability of intraday analysis ranged from RSD 0.13% to 1.23% (n = 3). The detection limit was 0.035 μg/mL, and the quantification limit was 0.117 μg/mL.

### 3.5. Degradation Kinetics Modeling

The reaction order of degradation of caffeic acid was obtained using the integral method [33]. This method uses a trial-and-error procedure to find reaction order. If the order assumed is correct, the appropriate plot of the concentration-time data [concentration against time (zero-order), lnconcentration against time (first-order), concentration^−1^ against time (second-order) and concentration^−2^ against time (third-order)] should be linear. The result showing the best correlation coefficient (R) was selected. The zero-order, first-order, second-order, third-order models are as follows in turn:

(2) $$c/c_{0}=-kt$$

(3) $$\ln c/c_{0}=-kt$$

(4) $$1/c-1/c_{0}=kt$$

(5) $$1/c^{2}-{1/c_{0}}^{2}=kt\;$$

where *c* is the concentration of reactant at given time, *c_0_* is the initial concentration of reactant, *k* is the rate constant, *t* is treatment time.

### 3.6. Determination of Degradation Products by FT-IR Spectroscopy

The functional groups of the degradation products at −5 to 25 °C were analyzed by FT-IR spectroscopy. FT-IR spectra of degradation products were obtained on a Nicolet 5700 (Thermo Fisher Scientific, Waltham, MA, USA). The wavenumber range covered was from 400–4,000 cm^−1^. The spectral resolution was 1 cm^−1^ and the collection time was about 1 min. The peaks analysis of FT-IR was performed with Origin-lab 7.5.

### 3.7. Determination of Degradation Products by HPLC-UV-ESIMS

The HPLC-UV-ESIMS system analyses were carried out on an Agilent-6460 Triple Quad LC/MS fitted with an ESI source. The HPLC conditions were in accordance with the description above with some revisions. Solvent A was changed to water containing 0.1% formic acid. Data acquisition and processing were performed using the Mass Hunter software. Positive and negative ion mass spectra of the column eluate were recorded in the range *m/z* 50–1,000. Fragmentor was set at 135 V, gas temperature was set at 325 °C and gas flow was 5 L/min. Sheath gas temperature was 250 °C and sheath gas flow was 11 L/min. The nozzle voltage was 500 V. Capillary voltage was 4,000 V for positive ion mode and −3,500 V for negative ion mode.

### 3.8. Statistical Analysis

Each treatment was replicated in triplicate. The results were expressed as mean ± SD. All the data were subjected to statistical analyses using SPSS16.0. The main effect of each factor of ultrasound treatment was subjected to analysis of variance and Duncan’s multiple range tests using One-Way ANOVA procedure. Mean values were considered significantly different when *p* < 0.05.

## 4. Conclusions

To obtain greater knowledge about the stability of caffeic acid during ultrasound-assisted extraction, the factors effecting degradation, the degradation kinetics and degradation products were investigated in a model system. The results indicated that caffeic acid was degraded under ultrasound treatment, and the type of solvents and temperature were important factors in determining the degradation reaction outcome and other factors such as liquid height, ultrasonic intensity and duty cycle of ultrasound exposure only affected the rate of degradation but did not change the nature of degradation. Degradation rate of caffeic acid decreased with increasing temperature. Degradation kinetics of caffeic acid under ultrasound fitted a zero–order reaction equation at −5 to 25 °C. Caffeic acid underwent decomposition and polymerization reactions under ultrasound. This study provides very useful information for the application of the ultrasound technique for the extraction of caffeic acid.

## References

[1] Sun-Waterhouse D., Zhou J., Miskelly G.M., Wibisono R., Wadhwaa S.S. (2011). Stability of encapsulated olive oil in the presence of caffeic acid. Food Chem..

[2] Stanely Mainzen Prince P., Senthil Kumaran K. (2012). Preventive effects of caffeic acid on lipids, lipoproteins and glycoproteins in isoproterenol induced myocardial infarcted rats. Food Res. Int..

[3] Göçer H., Gülçin Ì. (2011). Caffeic acid phenethyl ester (CAPE): Correlation of structure and antioxidant properties. Int. J. Food Sci. Nutr..

[4] Gulcin I. (2006). Antioxidant activity of caffeic acid (3,4-dihydroxycinnamic acid). Toxicology.

[5] Sanchez-Maldonado A.F., Schieber A., Gaenzle M.G. (2011). Structure-function relationships of the antibacterial activity of phenolic acids and their metabolism by lactic acid bacteria. J. Appl. Microbiol..

[6] Rehman M.U., Sultana S. (2011). Attenuation of oxidative stress, inflammation and early markers of tumor promotion by caffeic acid in Fe-NTA exposed kidneys of Wistar rats. Mol. Cell. Biochem..

[7] Patil S., Torres B., Tiwari B.K., Wijngaard H.H., Bourke P., Cullen P.J., O’Donnell C.P., Valdramidis V.P. (2010). Safety and quality assessment during the ozonation of cloudy apple juice. J. Food Sci..

[8] Vignoli J.A., Bassoli D.G., Benassi M.T. (2011). Antioxidant activity, polyphenols, caffeine and melanoidins in soluble coffee: The influence of processing conditions and raw material. Food Chem..

[9] Moon J.K., Shibamoto T. (2010). Formation of Volatile chemicals from thermal degradation of less volatile coffee components: Quinic acid, caffeic acid, and chlorogenic acid. J. Agric. Food Chem..

[10] Jiang D., Peterson D.G. (2010). Role of hydroxycinnamic acids in food flavor: A brief overview. Phytochem. Rev..

[11] Liazid A., Palma M., Brigui J., Barroso C.G. (2007). Investigation on phenolic compounds stability during microwave-assisted extraction. J. Chromatogr. A.

[12] Chandrapala J., Oliver C.M., Kentish S., Ashokkumar M. (2013). Use of power ultrasound to improve extraction and modify phase transitions in food processing. Food Rev. Int..

[13] Chemat F., Vian M.A., Cravotto G. (2012). Green extraction of natural products: Concept and principles. Int. J. Mol. Sci..

[14] Shirsath S.R., Sonawane S.H., Gogate P.R. (2012). Intensification of extraction of natural products using ultrasonic irradiations: A review of current status. Chem. Eng. Process..

[15] Paniwnyk L., Beaufoy E., Loyimer J.P., Mason T.J. (2001). The extraction of rutin from flower buds of *Sophora japonica*. Ultrason. Sonochem..

[16] Sun Y.J., Ma G.P., Ye X.Q., Kakuda Y., Meng R.F. (2010). Stability of all-*trans*-β-carotene under ultrasound treatment in a model system: Effects of different factors, kinetics and newly formed compounds. Ultrason. Sonochem..

[17] Iida Y., Tuziuti T., Yasui K., Towata A., Kozuka T. (2008). Control of viscosity in starch and polysaccharide solutions with ultrasound after gelatinization. Innov. Food Sci. Emerg. Technol..

[18] Gülseren İ., Güzey D., Bruce B.D., Weiss J. (2007). Structural and functional changes in ultrasonicated bovine serum albumin solutions. Ultrason. Sonochem..

[19] Ma Y.Q., Ye X.Q., Fang Z.X., Chen J.C., Xu G.H., Liu D.H. (2008). Phenolic compounds and antioxidant activity of extracts from ultrasonic treatment of satsuma mandarin (*Citrus unshiu* Marc.) peels. J. Agric. Food Chem..

[20] Atanassova D., Kefalas P., Petrakis C., Mantzavinos D., Kalogerakis N., Psillakis E. (2005). Sonochemical reduction of the antioxidant activity of olive mill wastewater. Environ. Int..

[21] Stalikas C.D. (2007). Extraction, separation, and detection methods for phenolic acids and flavonoids. J. Sep. Sci..

[22] Khan M.K., Abert-Vian M., Fabiano-Tixier A.S., Dangles O., Chemat F. (2010). Ultrasound-assisted extraction of polyphenols (flavanone glycosides) from orange (*Citrus sinensis* L.) peel. Food Chem..

[23] Wang A.Y., Zhou M.Y., Lin W.C. (2011). Antioxidative and anti-inflammatory properties of *Citrus sulcata* extracts. Food Chem..

[24] Ahmad J., Langrish T.A.G. (2012). Optimisation of total phenolic acids extraction from mandarin peels using microwave energy: The importance of the maillard reaction. J. Food Eng..

[25] Arend D.P., Santos T.C.D., Sonaglio D., Santos A.L.G., Reginatto F.H., Campos A.M.D. (2011). Experimental design as a tool to evaluate chlorogenic and caffeic acids extracted from *Cecropia glaziovii* Sneth. J. Pharm. Biomed. Anal..

[26] Hemwimol S., Pavasant P., Shotipruk A. (2006). Ultrasound-assisted extraction of anthraquinones from roots of *Morinda citrifolia*. Ultrason. Sonochem..

[27] Romdhane M., Gourdon C., Casamatta G. (1995). Local investigation of some ultrasonic devices by means of a thermal sensor. Ultrasonics.

[28] Kanthale P.M., Gogate P.R., Pandit A.B., Wilhelm A.M. (2003). Mapping of an ultrasonic horn: Link primary and secondary effects of ultrasound. Ultrason. Sonochem..

[29] Ma Y.Q., Ye X.Q., Hao Y.B., Xu G.H., Liu D.H. (2008). Ultrasound-assisted extraction of hesperidin from Penggan (*Citrus reticulata*) peel. Ultrason. Sonochem..

[30] Entezari M.H., Nazary S.H., Khodaparast M.H.H. (2004). The direct effect of ultrasound on the extraction of date syrup and its micro-organisms. Ultrason. Sonochem..

[31] Raso J., Maňas P., Pagán R., Sala F.J. (1999). Influence of different factors on the output power transferred into solvent by ultrasound. Ultrason. Sonochem..

[32] Xu G.H., Chen J.C., Liu D.H., Zhang Y.H., Jiang P., Ye X.Q. (2008). Minerals, phenolic compounds, and antioxidant capacity of citrus peel extract by hot water. J. Food Sci..

[33] Sun Y.J., Liu D.H., Chen J.C., Ye X.Q., Yu D. (2011). Effects of different factors of ultrasound treatment on the extraction yield of the all-*trans*-β-carotene from *Citrus* peels. Ultrason. Sonochem..

